# A new approach to evaluate linear programming problem in pentagonal neutrosophic environment

**DOI:** 10.1007/s40747-020-00181-0

**Published:** 2020-07-30

**Authors:** Sapan Kumar Das, Avishek Chakraborty

**Affiliations:** 1 Department of Revenue, Ministry of Finance, Government of India, New Delhi, India; 2 Department of Basic Science, Narula Institute of Technology, Agarpara, Kolkata India

**Keywords:** Linear Programming Problem, PNN, PNLP, Ranking Function, CrLP

## Abstract

In this paper, authors disclose a new concept of pentagonal neutrosophic (PN) approach to solve linear programming (LP) problem. To best of our insight, there is no approach for solving PNLP problem. For the first time, we take up the PNLP problem where the objectives, constraints are considered as pentagonal neutrosophic numbers (PNN). To deign our algorithm, we described the PN arithmetic operation laws and mathematical computation in PNN environment. This proposed method is based on ranking function and convert to its equivalent crisp LP (CrLP) problem. The obtained CrLP issue is presently being tackled by any LP method which is effectively accessible. To legitimize the proposed technique, some numerical tests are given to show the adequacy of the new model.

## Introduction

Prof. Zadeh [1] pioneered the idea of fuzzy set (F.S) in 1965, since then, researchers established pentagonal [2], hexagonal [3], heptagonal [4] F.S and their application in several arenas. Many researcher take pentagonal fuzzy number with different types of membership function. In this subsection we study on some published work which is associated with pentagonal fuzzy number. Sudha et al. [5] proposed a method for solving linear programming problem having pentagonal fuzzy numbers by using ranking function. Raj and Karthik [6] introduced the application of pentagonal fuzzy number in neural network. Some basic concept of pentagonal fuzzy number was proposed by Kamble [7]. Different mathematicians have been studying in different properties and application of pentagonal fuzzy numbers, see [8–10]. Panda and Pal [11] introduced a pentagonal fuzzy number method and appied in fuzzy matrix theory. Pathinathan and Ponnivalavan [12] introduced the reverse order linear membership function and define arithmetic operation on pentagonal fuzzy numbers. Garcia and Hernandez [48] proposed a interval type-2 fuzzy constraints applied in LP. A degree of satisfaction or possibility degree method was proposed by Srinivasan and Geetharamani [49] for solving type-2 fuzzy LP problem. Type-2 fuzzy is an emerging alternate representations of uncertainty instead of interval fuzzy and intuitionistic fuzzy set. At the point when the requirements of the issue are characterized by the assessment of different specialists or they depend on non-probabilistic data, the issue is the secret to quantify those suppositions and phonetic decisions, and afterward attempt to explain the issue. Since 1960’s, another sort of vulnerability called etymological vulnerability has been characterized. In this, the vulnerability about various view of an idea, for the most part given by numerous specialists with similarly important suppositions influences the meaning of the limitations of a LP issue. This sort of vulnerability can be tended to utilizing Interval Type-2 Fuzzy Sets (IT2FS). An IT2FS is a progressively mind boggling measure, so it needs more unpredictable portrayals than classical type fuzzy sets. Instead of structural expansion of F.S Further, Prof. Atanassov [13] in 1986 manifested the thought of intuitionistic fuzzy set (IFS) which is congenial mixture of membership and non-membership function. After that, Liu & Yuan [14] proposed triangular and Ye [15] explained the design of trapezoidal IFS and Several brilliant works has been issued in this F.S and IFS ground till now. Later, in 1998 Prof. F. Smarandache [16] proposed the structure of neutrosophic set (NS) and development of NS can grab any kind of realistic problem in a logical way. Further, Wang et al. [17, 18] in 2010 manifested the design of single typed neutrosophic set which plays an essential role in NS theory.

Recently, in 2018–2019 Chakraborty et al. [19, 20] germinated the perception of triangular and trapezoidal neutrosophic number respectively and its categorization according to the dependency of the membership function. Also, in 2019, Chakraborty et al. [21] promoted the concept of pentagonal neutrosophic number (PNN) and further, established the knowledge of Crispification [22] of PNN in research domain. Later, Chakraborty et al. [23] introduced the idea of aggregation operator on PNN and applied it in MCGDM section, networking field and graph theory problem [24, 25].Several articles are created in neutron-logic domain like, Basset et al. [26] proposed type- 2 NS, Deli et al. [27] pioneered the thought of bipolar NS, Nabeeh et al. [28] categorized neutrosophic AHP skill, Chakraborty et al. [29, 30] initiated cylindrical neutrosophic logic, Pal [31] created EOQ model using NS, Haque [32] suggested generalized spherical set concept etc. Recently, numerous articles are published in netro-logic based operation research problem like Hariri and Ata [33] germinated GP approach to solve inventory model varying order cost, Jung and Klein [34, 35] manifested comparative study between the total cost minimization and the profit maximization model, Mandal et al. [36] introduced inventory model of declined items utilizing GP method, Leung [37] manifested an EPQ model having flexible and imperfect production method using GP approach, Wakeel et al. [38] proposed multi-product, multi-vendors inventory models under linear and non-linear constraints, Roy and Das [39] resolved multi objective production planning problem in neutro-logic arena.

Neutrosophic hypothesis applied in numerous fields of sciences, so as to take care of the issues identified with indeterminacy. In like manner, Abdel-Basset [40] added the neutrosophic LP models the place their parameters are tended with trapezoidal neutrosophic numbers and introduced a method for getting them. Das and Jatindra [41] introduced a strategy for solving neutrosophic LP problem having triangular neutrosophic numbers by using ranking function. Edalatpanah [42] presented a direct aproach of neutrosophic LP problem having triangular fuzzy number. Again, Edalatpanah [43] presented a aggregate ranking function of data envelopment analysis based on triangular neutrosophic numbers. In M. Mohamed et al., [44] introduced another score function for neutrosophic integer programming problems having triangular neutrosophic numbers. Banerjee and Pramanik [45] added the LP problem with single objective in neutrosophic number (NN) conditation with the assistance of goal programming. Maiti et al. [46] introduced a strategy for multi-level-multi-objective LP problem by the assistance of goal programming. Hussian et al. [47] proposed a neutrosophic LP issue using ranking function. Several works have been published recently [50–60] in uncertainty theory which plays an important role in vagueness research world.

### Contribution

Traditional LP issues include deterministic target works and constrained functions. In any case, in our mind there is consistently possibility of vulnerability, all things considered, issue. Because of blunder in estimating method, instrumental brokenness, and so forth., a few information as we would see it can’t be correctly, truth or precisely decided. This concept of variation leads a new type of neutrosophic numbers called pentagonal neutrosophic numbers.

The main advantage of neutrosophic set is that it’s help the decision-makers making by considering truth degree, falsity degree and indeterminacy degree. Here indeterminacy degree is for the most part considered as a free factor which has a significant commitment in decision-making. Due to some uncertainty in real world problem, it is better to use pentagonal NLP problem instead of classical PLP problem. For avoiding the unrealistic modelling we used PNLP model in practical situations. Supposedly, no one considered the neutrosophic vulnerability in the factors for LP models having PNN. In this article, a NLP issue is thought of, where all the coefficients are thought to be a pentagonal neutrosophic numbers. Along these lines, we are proposing a calculation for taking care of PNLP issue with the assistance of the newly developed ranking function. Utilizing the newly ranking function, the PNLP issue is changing over into its identical crisp LP issue. To best of our knowledge, it would be the first method to solve the PNLP problem. Thus, for the approval of created technique, direct correlation with relative strategies doesn’t emerge. Along these lines, we contrast our proposed technique with some current fuzzy LP issue [5]. Another Diet outline issue is delineated to show the effectiveness and utilization of our technique, in actuality, issue.

### Motivation

Neutrosophic sets plays an important role in uncertainty modelling. The development of uncertainty theory plays a fundamental role in formulation of real-life scientific mathematical model, structural modelling in engineering field, medical diagnoses problem etc. Recently, a question will come up, how can we convert a PNN equivalent to a crisp number in logical and scientific way? Several works has been already published in PNN arena till now and the need of Crispification is also explained in dissimilar articles. How can we implement it in a linear programming based operation research problem? Is it possible to apply in real life problem? Still there is no method for applying in linear programming problem having pentagonal neutrosophic numbers. From this aspect we try to extend this research article.

### Novelties

In this current decade, researchers have exposed their considerations to make progress with the theories related to neutrosophic area and constantly try to endorse its sufficient scope applications in dissimilar branches of neutrosophic domain. However, justifying all the views connecting to PNN theory our main objective is to support the theory efficiently with these following points.

1. Introduced new score function and its efficiency.
2. Application of PNN in linear programming problem.
3. Compared the results with previous established results.

## Mathematical preliminaries

### **Definition 2.1**

**(**Fuzzy Set (F.S): [1]) ${\tilde{L}}$ is identified as a fuzzy set, for the pair $\left( {x,\mu_{{\tilde{L}}} \left( x \right)} \right)$ it can be written as $\tilde{L} = \left\{ {\left( {x,\mu_{{\tilde{L}}} \left( x \right)} \right):x \in X,\mu_{{\tilde{L}}} \left( X \right) \in \left[ {0,1} \right]} \right\}$ where $x$ $\in X$ and $\mu_{{\tilde{L}}} \left( X \right)$ $\in \left[ {0,1} \right].$

### **Definition 2.2**

(Intuitionistic Fuzzy Set (IFS): [13]) $\tilde{S}_{\text{F}}$ is identified as an Intuitionistic set if $\tilde{S}_{\text{F}} = \left\{ {x;\left[ {\varphi \left( x \right),\omega \left( x \right)} \right] \vdots x \in X = {\text{Universal}}\; {\text{set}}} \right\}$, where $\varphi \left( x \right):X \to \left[ {0,1} \right]$ is termed as membership function, $\omega \left( x \right):X \to \left[ {0,1} \right]$ is termed as non-membership function.

$\varphi \left( x \right), \omega \left( x \right)$ exhibits the following relation $0 \le \varphi \left( x \right) + \omega \left( x \right) \le 1$.

### **Definition 2.3**

(Neutrosophic Set (NS): [16]) A set $\widetilde{\text{NEU}}_{\text{M}}$ is identified as a neutrosophic set if $\widetilde{\text{NEU}}_{\text{M}} = \left\{ {x;\left[ {\theta_{{\widetilde{\text{NEU}}_{\text{M}} }} \left( x \right),\varphi_{{\widetilde{\text{NEU}}_{\text{M}} }} \left( x \right),\sigma_{{\widetilde{\text{NEU}}_{\text{M}} }} \left( x \right)} \right] \vdots x \in X} \right\}$, where $\theta_{{\widetilde{\text{NEU}}_{\text{M}} }} \left( x \right):X \to \left] { - 0,1 + } \right[$ is declared as the truthness function, $\varphi_{{\widetilde{\text{NEU}}_{\text{M}} }} \left( x \right):X \to \left] { - 0,1 + } \right[$ is declared as the hesitation function, and $\sigma_{{\widetilde{\text{NEU}}_{\text{M}} }} \left( x \right):X \to \left] { - 0,1 + } \right[$ is declared as the falseness function.

$\theta_{{\widetilde{\text{NEU}}_{\text{M}} }} \left( x \right), \varphi_{{\widetilde{\text{NEU}}_{\text{M}} }} \left( x \right) \;{\text{and}}\; \sigma_{{\widetilde{\text{NEU}}_{\text{M}} }} \left( x \right)$ displays the following relation:

$$ - 0 \le {\text{Sup }}\{ \theta_{{\widetilde{\text{NEU}}_{\text{M}} }} \left( x \right)\} + {\text{Sup}} \{ \varphi_{{\widetilde{\text{NEU}}_{\text{M}} }} \left( x \right)\} + {\text{Sup}} \{ \sigma_{{\widetilde{\text{NEU}}_{\text{M}} }} \left( x \right)\} \le 3 + $$

### **Definition 2.4**

(Single-Valued Neutrosophic Set (SNS): [17, 18]) A set $\widetilde{\text{NEU}}_{\text{M}}$ in the definition 2.3 is called as a SNS $\left( {\widetilde{\text{SNEU}}_{\text{M}} } \right)$ if $x$ is a single-valued independent variable. $\widetilde{\text{SNEUA}} = \left\{ {x;\left[ {\theta_{{\widetilde{\text{SNEU}}_{\text{M}} }} \left( x \right),\varphi_{{\widetilde{\text{SNEU}}_{\text{M}} }} \left( x \right),\sigma_{{\widetilde{\text{SNEU}}_{\text{M}} }} \left( x \right)} \right] \vdots x \in X} \right\}$, $\theta_{{\widetilde{\text{SNEU}}_{\text{M}} }} \left( x \right), \varphi_{{\widetilde{\text{SNEU}}_{\text{M}} }} \left( x \right)\;{\text{and}}\; \sigma_{{\widetilde{\text{SNe}}_{\text{M}} }} \left( x \right)$ signified the notion of correct, indefinite and incorrect memberships function respectively.

### **Definition 2.5**

(Single-Valued Pentagonal Neutrosophic Number (SPNN): [21]) A SPNN $\left( {\tilde{M}} \right)$ is defined as $\widetilde{\text{SPNEU}} = \left[ {\left( {m^{1} ,n^{1} ,o^{1} ,p^{1} ,q^{1} } \right);\mu } \right],\left[ {\left( {m^{2} ,n^{2} ,o^{2} ,p^{2} ,q^{2} } \right);\theta } \right],\left[ {\left( {m^{3} ,n^{3} ,o^{3} ,p^{3} ,q^{3} } \right);\eta } \right]$, where $\mu ,\theta ,\eta \in \left[ {0,1} \right]$. The truth membership function $\left( {\mu_{{\widetilde{\text{SPNEU}}}} } \right):{\mathbb{R}} \to \left[ {0,\mu } \right]$, the hesitant membership function $\left( {\theta_{{\widetilde{\text{SPNEU}}}} } \right):{\mathbb{R}} \to \left[ {\theta ,1} \right]$ and the false membership function $\left( {\eta_{{\widetilde{\text{SPNEU}}}} } \right):{\mathbb{R}} \to \left[ {\eta ,1} \right]$ are given as:

$$ \begin{aligned} \mu_{{\widetilde{\text{SPNEU}}}} \left( x \right) = \left\{ {\begin{array}{*{20}c} {\mu_{{\widetilde{\text{Ssl1}}}} \left( x \right)} & {m^{1} \le x < n^{1} } \\ {\mu_{{\widetilde{\text{Ssl2}}}} \left( x \right)} & {n^{1} \le x < o^{1} } \\ \mu & {x = o^{1} } \\ {\mu_{{\widetilde{\text{Ssr2}}}} \left( x \right)} & {o^{1} \le x < p^{1} } \\ {\mu_{{\widetilde{\text{Ssr1}}}} \left( x \right)} & {p^{1} \le x < q^{1} } \\ 0 & {\text{otherwise}} \\ \end{array} ,} \right.\quad \theta_{{\widetilde{\text{SPNEU}}}} \left( x \right) = \left\{ {\begin{array}{*{20}c} {\theta_{{\widetilde{\text{Ssl1}}}} \left( x \right)} & {m^{2} \le x < n^{2} } \\ {\theta_{{\widetilde{\text{Ssl2}}}} \left( x \right)} & {n^{2} \le x < o^{2} } \\ \vartheta & {x = o^{2} } \\ {\theta_{{\widetilde{\text{Ssr2}}}} \left( x \right)} & {o^{2} \le x < p^{2} } \\ {\theta_{{\widetilde{\text{Ssr1}}}} \left( x \right)} & {p^{2} \le x < q^{2} } \\ 1 & {\text{otherwise}} \\ \end{array} } \right. \hfill \\ \eta_{{\widetilde{\text{SPNEU}}}} \left( x \right) = \left\{ {\begin{array}{*{20}c} {\eta_{{\widetilde{\text{Ssl1}}}} \left( x \right)} & {m^{3} \le x < n^{3} } \\ {\eta_{{\widetilde{\text{Ssl2}}}} \left( x \right)} & {n^{3} \le x < o^{3} } \\ \vartheta & {x = o^{3} } \\ {\eta_{{\widetilde{\text{Ssr2}}}} \left( x \right)} & {o^{3} \le x < p^{3} } \\ {\eta_{{\widetilde{\text{Ssr1}}}} \left( x \right)} & {p^{3} \le x < q^{3} } \\ 1 & {\text{otherwise}} \\ \end{array} } \right. \hfill \\ \end{aligned} $$

## Score and accuracy function

Let us consider a single valued PNN as $\tilde{F}_{\text{Pen}} = \left( {F_{1} ,F_{2} ,F_{3} ,F_{4} ,F_{5} ;\pi ,\sigma ,\rho } \right)$, The primary application of score function is to drag the judgment of conversion of PNN to crisp number. Also, the mean of the PNN components is $\frac{{\left( {F_{1} + F_{2} + F_{3} + F_{4} + F_{5} } \right)}}{5}$ and score value of the membership portion is $\frac{{\left\{ {2 + \pi - \rho - \sigma } \right\}}}{3}$.

Thus, for a P.N.N $$ \tilde{F}_{\text{Pen}} = \left( {F_{1} ,F_{2} ,F_{3} ,F_{4} ,F_{5} ;\pi ,\sigma ,\rho } \right) $$,

Score function is scaled as $\widetilde{\text{SC}}_{\text{Pen}} = \frac{1}{15}\left( {F_{1} + F_{2} + F_{3} + F_{4} + F_{5} } \right) \times \left\{ {2 + \pi - \rho - \sigma } \right\}$

Accuracy function is scaled as $\widetilde{\text{AC}}_{\text{Pen}} = \frac{1}{15}\left( {F_{1} + F_{2} + F_{3} + F_{4} + F_{5} } \right) \times \left\{ {2 + \pi - \sigma } \right\}$

Here, $$ \widetilde{\text{SC}}_{\text{Pen}} \in R $$, $$ \widetilde{\text{AC}}_{\text{Pen}} \in R $$

### Relationship between any two PNN

Let us consider any two pentagonal neutrosophic number defined as follows

$$ F_{\text{Pen1}} = \left( {\pi_{\text{Pen1}} ,\sigma_{\text{Pen1}} ,\rho_{\text{Pen1}} } \right) ,\;F_{Pen2} = \left( {\pi_{\text{Pen2}} ,\sigma_{\text{Pen2}} ,\rho_{\text{Pen2}} } \right) $$

1. $$ {\text{SC}}_{\text{Pen1}} > {\text{SC}}_{\text{Pen2}} , F_{\text{Pen1}} > F_{\text{Pen2}} $$
2. $$ {\text{SC}}_{\text{Pen1}} < {\text{SC}}_{\text{Pen2 }} , F_{\text{Pen1}} < F_{\text{Pen2}} $$
3. $$ {\text{SC}}_{\text{Pen1}} = {\text{SC}}_{\text{Pen2}} ,F_{\text{Pen1}} = F_{\text{Pen2}} $$

Then,

$$ {\text{AC}}_{\text{Pen1}} > {\text{AC}}_{\text{Pen2}} , F_{\text{Pen1}} > F_{\text{Pen2}} $$

$$ {\text{AC}}_{\text{Pen1}} < {\text{AC}}_{\text{Pen2 }} , F_{\text{Pen1}} < F_{\text{Pen2}} $$

$$ {\text{AC}}_{\text{Pen1}} = {\text{AC}}_{\text{Pen2}} ,F_{\text{Pen1}} = F_{\text{Pen2}} $$

## Basic operations

Let $\tilde{F}_{1}$  = 〈($c_{1} , c_{2}$, $c_{3}$, $c_{4}$, $c_{5}$); $\pi_{{\tilde{p}_{1} }}$, $\mu_{{\tilde{p}_{1} }}$, $\sigma_{{\tilde{p}_{1} }}$〉 and $\tilde{F}_{2}$  = 〈($d_{1} , d_{2}$, $d_{3}$, $d_{4}$, $d_{5}$); $\pi_{{\tilde{p}_{2} }}$, $\mu_{{\tilde{p}_{2} }}$, $\sigma_{{\tilde{p}_{2} }}$〉 be two IPFNs and $\alpha \ge 0$. Then the following operational relations hold:

1. $$ \tilde{F}_{1} $$  +  $$ \tilde{F}_{2} $$  = 〈($$ c_{1} $$  +  $$ d_{1} $$, $$ c_{2} $$  +  $$ d_{2} $$, $$ c_{3} $$  +  $$ d_{3} $$, $$ c_{4} $$  +  $$ d_{4} $$, $$ c_{5} $$  +  $$ d_{5} $$); $$ { \hbox{max} }\{ \pi_{{\tilde{p}_{1} }} ,\pi_{{\tilde{p}_{2} }} \} $$, $$ { \hbox{min} }\{ \mu_{{\tilde{p}_{1} }} $$ $$ ,\mu_{{\tilde{p}_{2} }} \} $$,$$ \hbox{min} \{ \sigma_{{\tilde{p}_{1} }} $$ $$ ,\sigma_{{\tilde{p}_{2} }} \} $$ 〉
2. $$ \tilde{F}_{1} $$  −  $$ \tilde{F}_{2} $$  = 〈($$ c_{1} $$  −  $$ d_{5} $$, $$ c_{2} $$  −  $$ d_{4} $$, $$ c_{3} $$  −  $$ d_{3} $$, $$ c_{4} $$  −  $$ d_{2} $$, $$ c_{5} $$  −  $$ d_{1} $$); $$ { \hbox{max} }\{ \pi_{{\tilde{p}_{1} }} ,\pi_{{\tilde{p}_{2} }} \} $$, $$ { \hbox{min} }\{ \mu_{{\tilde{p}_{1} }} $$ $$ ,\mu_{{\tilde{p}_{2} }} \} $$, $$ \hbox{min} \{ \sigma_{{\tilde{p}_{1} }} $$ $$ ,\sigma_{{\tilde{p}_{2} }} \} $$ 〉
3. $$ \tilde{F}_{1} \times \tilde{F}_{2} $$  = 〈($$ c_{1} d_{1} $$, $$ c_{2} d_{2} , c_{3} d_{3} $$, $$ c_{4} d_{4} $$, $$ c_{5} d_{5} $$); $$ \pi_{{\tilde{p}_{1} }} $$$$ \pi_{{\tilde{p}_{2} }} $$, $$ \mu_{{\tilde{p}_{1} }} + , \mu_{{\tilde{p}_{2} }} - \mu_{{\tilde{p}_{1} }} $$ $$ \mu_{{\tilde{p}_{2} }} $$, $$ \sigma_{{\tilde{p}_{1} }} + \sigma_{{\tilde{p}_{2} }} - \sigma_{{\tilde{p}_{1} }} \sigma_{{\tilde{p}_{2} }} $$〉
4. $$ \alpha \tilde{F}_{1} $$   = 〈$$ (\alpha c_{1} $$, $$ \alpha c_{2} $$, $$ \alpha c_{3} $$, $$ \alpha c_{4} $$, $$ \alpha c_{5} $$); 1 $$ - \left( {1 - \pi_{{\tilde{p}_{1} }} } \right)^{\alpha } ,\mu_{{\tilde{p}_{1} }}^{\alpha } ,\sigma_{{\tilde{p}_{1} }}^{\alpha } $$)〉
5. $$ \tilde{F}_{1}^{\alpha } $$  = 〈($$ c_{1}^{\alpha } $$, $$ c_{2}^{\alpha } ,c_{3}^{\alpha } $$, $$ c_{4}^{\alpha } , c_{5}^{\alpha } $$); $$ \pi_{{\tilde{p}_{1} }}^{\alpha } $$, $$ \left( {1 - \mu_{{\tilde{p}_{1} }} } \right)^{\alpha } , \left( {1 - \sigma_{{\tilde{p}_{1} }} } \right)^{\alpha } $$〉

## Neutrosophic linear programming problem

Consider the standard form of neutrosophic linear programming (NLP) problem with *m* constraints and *n* variables having all coefficients and resources are represented pentagonal neutrosophic numbers as follows:

1 $$ \begin{aligned} \,\,\,\,\,\,\,\,\,\,\,\,\,\,\,\,\,\,\,\,\,\,\,\,\,{\text{Max}}\, ( {\text{Min)}}\,\,(\tilde{c}^{t} x) \hfill \\ {\text{subject}}\,{\text{to}} \hfill \\ & \,\,\,\,\,\,\,\,\,\,\,\,\,\,\,\,\,\tilde{A}x \le \tilde{b}, \hfill \\ \end{aligned} $$

where, $x$ is a non-negative number.

The steps of the proposed method are as follows:

***Step 1:*** Use instead of $\tilde{c}^{t} = [\tilde{c}_{j} ]_{1 \times n} ,$ $\tilde{A} = [\tilde{a}_{ij} ]_{m \times n} ,$ $\tilde{b} = [b_{i} ]_{m \times 1}$ then the problem (1) may be written

2 $$ {\text{as}}:{\text{ Max }}\left( {\text{Min}} \right)\sum\limits_{j = 1}^{n} {\tilde{c}_{j} x} $$

Subject to, $\sum\limits_{j = 1}^{n} {\tilde{a}_{ij} } x = \tilde{b}_{i}$ for all *i* = 1,2,…,*m*.

$x$ is a non-negative number.

***Step 2:*** If the parameters of $\tilde{c}_{j} ,$ $\tilde{a}_{ij} ,$ and $\tilde{b}_{i}$ are considered as pentagonal neutrosophic numbers 〈($c_{1} , c_{2}$, $c_{3}$, $c_{4}$, $c_{5}$); $\pi_{{\tilde{p}_{1} }}$, $\mu_{{\tilde{p}_{1} }}$,$\sigma_{{\tilde{p}_{1} }}$〉, 〈$(a_{11} ,a_{22} ,a_{33} ,a_{44} ,a_{55} ); \, \pi_{{\tilde{p}_{1} }} ,\mu_{{\tilde{p}_{1} }} ,\sigma_{{\tilde{p}_{1} }}$〉 and 〈$(b_{1} ,b_{2} ,b_{3} ,b_{4} ,b_{5} );\pi_{{\tilde{p}_{1} }} ,\mu_{{\tilde{p}_{1} }} ,\sigma_{{\tilde{p}_{1} }}$〉 respectively, then the problem (2) may be written as:

3 $$ {\text{Max }}\left( {\text{Min}} \right)\;(c_{1} ,c_{2} ,c_{3} ,c_{4} ,c_{5} ;\pi_{{\tilde{p}_{1} }} ,\mu_{{\tilde{p}_{1} }} ,\sigma_{{\tilde{p}_{1} }} )x $$

Subject to, $$ (a_{11} ,a_{22} ,a_{33} ,a_{44} ,a_{55} ;\pi_{{\tilde{p}_{1} }} ,\mu_{{\tilde{p}_{1} }} ,\sigma_{{\tilde{p}_{1} }} )x \le (b_{1} ,b_{2} ,b_{3} ,b_{4} ,b_{5} ;\pi_{{\tilde{p}_{1} }} ,\mu_{{\tilde{p}_{1} }} ,\sigma_{{\tilde{p}_{1} }} ) $$

$x$ is a non-negative number.

***Step 3:*** As we consider pentagonal neutrosophic numbers $\tilde{p}_{\text{pen}} = (R_{1} ,R_{2} ,R_{3} ,R_{4} ,R_{5} ;\pi ,\rho ,\sigma )$ and the ranking function may be written as mathematically:

$$ \Re (\tilde{p}_{\text{pen}} ) = \frac{1}{15}(R_{1} + R_{2} + R_{3} + R_{4} + R_{5} )(2 + \hbox{min} \pi - \hbox{max} \rho - \hbox{max} \sigma ) $$

***Step 4:*** Applying our new ranking function to convert each pentagonal neutrosophic numbers to its equivalent crisp value. Now the problem obtained in Step 3, might be rewritten as:

4 $$ {\text{Max }}\left( {\text{Min}} \right)\Re (c_{1} ,c_{2} ,c_{3} ,c_{4} ,c_{5} ;\pi_{{\tilde{p}_{1} }} ,\mu_{{\tilde{p}_{1} }} ,\sigma_{{\tilde{p}_{1} }} )x $$

Subject to, $$ \Re (a_{11} ,a_{22} ,a_{33} ,a_{44} ,a_{55} ;\pi_{{\tilde{p}_{1} }} ,\mu_{{\tilde{p}_{1} }} ,\sigma_{{\tilde{p}_{1} }} )x \le \Re (b_{1} ,b_{2} ,b_{3} ,b_{4} ,b_{5} ;\pi_{{\tilde{p}_{1} }} ,\mu_{{\tilde{p}_{1} }} ,\sigma_{{\tilde{p}_{1} }} ) $$

$x$ is a non-negative number.

***Step 5:*** The problem obtained in Step 4, now the problem is crisp model and solves the crisp model using the standard method and obtains the optimal solution of problem.

### Flowchart


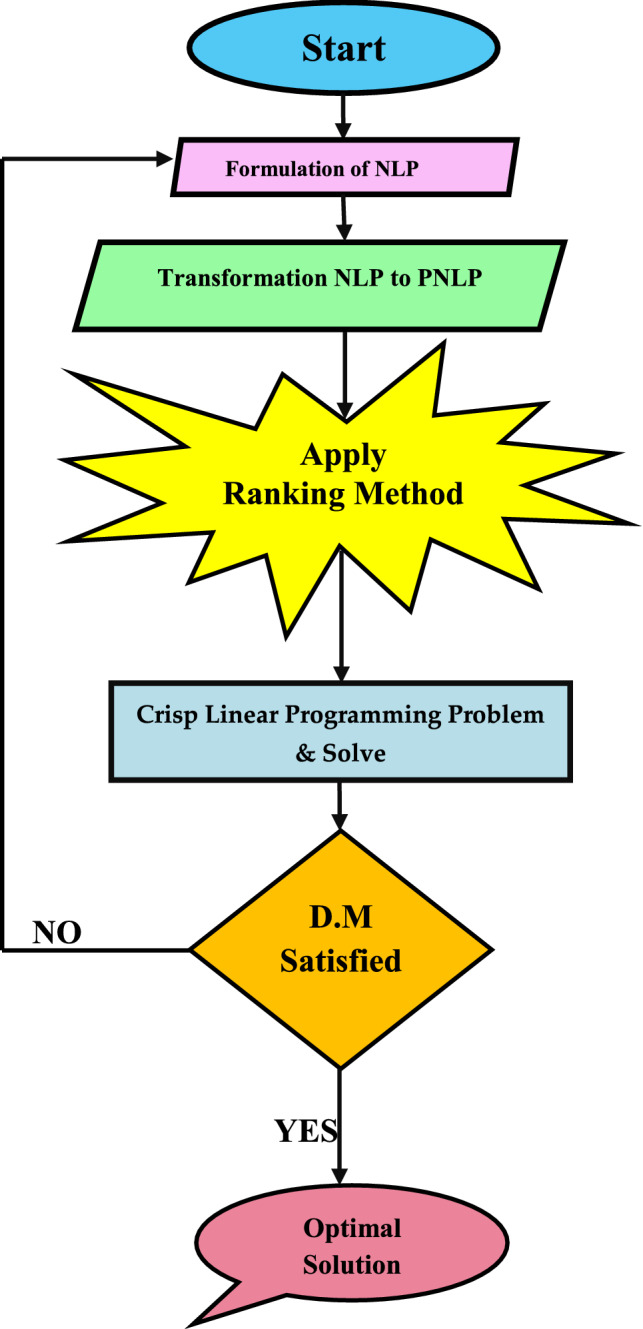


## Numerical examples

To best of our mind, still there is no method to solve PNLP problem, therefore, in this section we consider a new method to solve PNLP problem and compare with fuzzy pentagonal LP problem. To proved the applicability and efficiency of our proposed method, we consider the fuzzy problem which introduced by [5]. The main drawback in fuzzy pentagonal numbers is the manager only consider the truth degree, however, the neutrosophic pentagonal numbers is consider truth, indeterminacy and falsity degree. In real life problem, if the manager in-charge of the diet plans for high school launch. The main job of manager is to make sure that the students get the right balance of nutrition from the chosen food. However, there are some restrictions in terms of budget, class of students, available of items and the variety of food that needs to be in the diet to make it interesting. Therefore, in this case the manager wants to avoid the uncertainty, indeterminacy and truth value, he has applied the neutrosophic numbers instead of fuzzy for better outcome.

### *Example 1*

Consider the following fuzzy pentagonal numbers numerical example considered by [5].

$$ \hbox{max} = (0.8,0.7,0.5,0.3,0.2)x_{1} + (0.2,0.3,0.4,0.1,0.2)x_{2} $$

Subject to

$$ \begin{aligned} (0.2,0.4,0.5,0.6,0.7)x_{1} + (0.3,0.2,0.6,0.5,0.1)x_{2} \le (0.1,0.2,0.5,0.4,0.3) \hfill \\ (0.7,0.8,0.6,0.9,0.1)x_{1} + (0.2,0.3,0.5,0.7,0.1)x_{2} \le (0.2,0.3,0.5,0.7,0.9) \hfill \\ \end{aligned} $$

$$ x_{1} ,x_{2} \ge 0 $$

Now the problem is pentagonal fuzzy number. Therefore, the decision maker consider the confirmation degree of each pentagonal number is (1, 0, 0). Based on step 2, we convert the above problem into pentagonal neutrosophic numbers in the following format.

$$ \hbox{max} = (0.8,0.7,0.5,0.3,0.2;1,0,0)x_{1} + (0.2,0.3,0.4,0.1,0.2;1,0,0)x_{2} $$

Subject to, $$ \begin{aligned} (0.2,0.4,0.5,0.6,0.7;1,0,0)x_{1} + (0.3,0.2,0.6,0.5,0.1;1,0,0)x_{2} \le (0.1,0.2,0.5,0.4,0.3;1,0,0) \hfill \\ (0.7,0.8,0.6,0.9,0.1;1,0,0)x_{1} + (0.2,0.3,0.5,0.7,0.1;1,0,0)x_{2} \le (0.2,0.3,0.5,0.7,0.9;1,0,0) \hfill \\ \end{aligned} $$

$$ x_{1} ,x_{2} \ge 0 $$

By utilizing our step-3, new ranking function, the issue of PNLP problem is converting into crisp LP problem and the problem will be as follows:

$$ \hbox{max} = 0.5x_{1} + 0.34x_{2} $$

Subject to

$$ \begin{aligned} 0.48x_{1} + 0.34x_{2} \le 0.3 \hfill \\ 0.62x_{1} + 0.36x_{2} \le 0.52 \hfill \\ \end{aligned} $$

$$ x_{1} ,x_{2} \ge 0 $$

Here, we can solve the crisp LP problem by using LATEX 18.0 we get the optimal solution.

The solution is: $x_{1} = 0.625,x_{2} = 0$ and $Z = 0.3125$. Let us see in the following comparison table with existing method and its very crystal clear that our method is always maximizing the result as the decision maker wanted.

### *Example 2*

Consider the following fuzzy pentagonal numbers numerical example considered by [5].

$$ \hbox{max} = (11,13,15,17,19)x_{1} + (31,33,35,37,39)x_{2} $$

Subject to

$$ \begin{aligned} (41,43,45,47,49)x_{1} + (61,63,65,67,69)x_{2} \le (151,153,155,157,159) \hfill \\ (81,83,85,87,89)x_{1} + (101,103,105,107,109)x_{2} \le (271,273,275,277,279) \hfill \\ \end{aligned} $$

$$ x_{1} ,x_{2} \ge 0 $$

Now the problem is pentagonal fuzzy number. Therefore, the decision maker considers the different confirmation degree of each pentagonal number. Based on step 2, we convert the above problem into pentagonal neutrosophic numbers in the following format.

$$ \hbox{max} = (11,13,15,17,19;0.6,0.3,0.2)x_{1} + (31,33,35,37,39;0.7,0.1,0.4)x_{2} $$

Subject to

$$ \begin{aligned} (41,43,45,47,49;0.6,0.3,0.5)x_{1} + (61,63,65,67,69;0.8,0.4,0.6)x_{2} \le \hfill \\ (151,153,155,157,159;0.7,0,0.55) \hfill \\ (81,83,85,87,89;0.5,0.4,0.1)x_{1} + (101,103,105,107,109;0.8,0.45,0.3)x_{2} \le \hfill \\ (271,273,275,277,279;0.4,0.25,0.5) \hfill \\ \end{aligned} $$

$$ x_{1} ,x_{2} \ge 0 $$

By utilizing our step-3, new ranking function, the issue of PNLP problem is convert into crisp LP problem and the problem will be as follows:

$$ \hbox{max} = 10.5x_{1} + 25.67x_{2} $$

Subject to

$$ \begin{aligned} 27x_{1} + 39x_{2} \le 111.083 \hfill \\ 56.67x_{1} + 71.25x_{2} \le 151.25 \hfill \\ \end{aligned} $$

$$ x_{1} ,x_{2} \ge 0 $$

Here, we can solve the crisp LP problem by using LATEX 18.0 we get the optimal solution.

The solution is: $x_{1} = 0,x_{2} = 2.1229$ and $Z = 54.492$. Let us see in the following comparison table with existing method and its very crystal clear that our method is always maximizing the result as the decision maker wanted.

### Simulation and comparative study

**Fig. 1:**
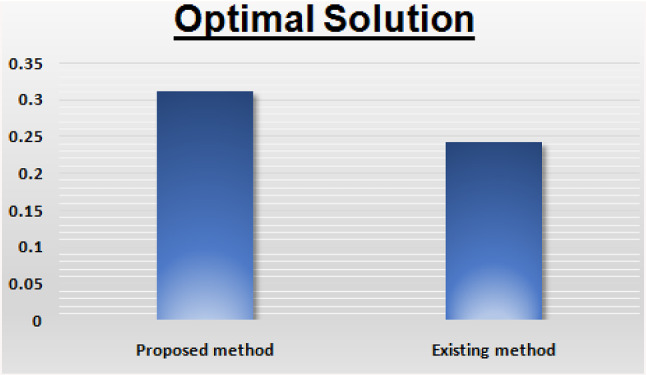
Graphical representation of comparison of work

**Fig. 2:**
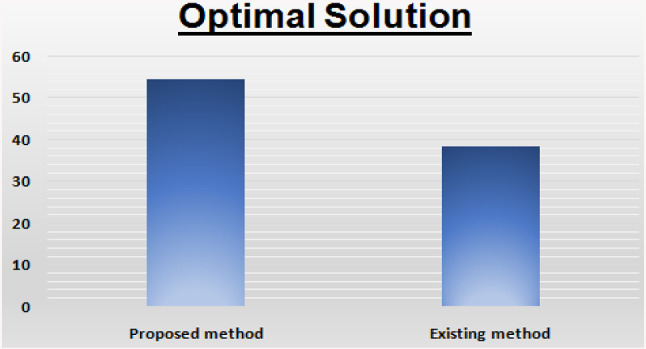
Graphical representation of comparison of work

This section provides a comparative study of the proposed algorithm with the existing method of for pentagonal neutrosophic linear programming problems. A comparison of the results between existing and new techniques is shown in Tables 1 and 2. In Figs. 1 and 2 (Graphical comparison with existing methods) when we have compared our proposed method with the other existing methods, we have found that the objective value of our proposed method is maximum than to the existing methods.

**Table 1:** Comparison with existing method: [5]

Solution	Proposed method	Existing method
Optimal value	$x_{1} = 0.625,x_{2} = 0$	$x_{1} = 0.526,x_{2} = 0$
Optimal solution	$Z = 0.3125$	$Z = 0.2428$

**Table 2:** Comparison with existing method [5]

Solution	Proposed method	Existing method
Optimal value	$x_{1} = 0,x_{2} = 2.1229$	$x_{1} = 0,x_{2} = 1.852$
Optimal solution	$Z = 54.492$	$Z = 38.24$

Simulation results of predicted optimum cost values with the existing methods (Table 3).

**Table 3:** Simulation and comparison table

	Proposed method		Existing method [5]	
	Elapsed run time seconds	Total Iteration	Elapsed run time seconds	Total Iteration
Example-1	0.04	02	0.05	02
Example-2	0.05	03	0.05	03

From the above comparison both simulation and graphical representations, we can conclude that our proposed method is superior to the existing method. Additionally, we can say that the objective value obtained by our proposed method lies within neutrosophic region.

### Advantages of proposed method

By analysis of proposed method with existing method [5] result of the same problem, we noted that:

1. Our proposed model results are better than Sudha results. Let us look at the optimal tableau of our proposed model as shown in Table 1, it is obvious that the objective function value equal 0.3125 but in Sudha, the objective function equal 0.2428 by knowing that, the problem is a maximization problem. To make this more clarity, let us look the optimum tableau as shown in Table 2, it conclude that the objective function is 54.492 however, in Sudha method the objective function is 38.24. As the problem is maximization and our proposed objective solution is also maximized.
2. In our daily life problem, the manager always facing to take decision in the form of agree, not sure and disagree. However, in Sudha fuzzy model the manager is the taking the decision of truthiness function only. This is the main drawback of Sudha fuzzy model. Therefore, we consider this drawback in our model and we using neutrosophic model.
3. Our model is more simple and efficient than Sudha model.
4. Our proposed model is applied in real-life problem and large scale problem.

In the above discussion, we can conclude that our proposed algorithm is a new way to handle the uncertainty and indeterminacy in the real-life problem.

In the following sub-section, we illustrate a real-life problem by using our proposed method.

### Real life problem

Recently, the outbreak of Covid-19 is spread all over the world. The decision maker still not confirm the medicine or vaccine for treatment the covid-19 patients. It is also not known to decision-maker, the life span of this virus. Only to improve the immunity system in our body is the main issue directed by WHO. Therefore, we solved a real life diet chart problem.

Below there is a diet chart that gives me calories, protein and carbohydrate content for 3 food items with three products like Banana, Apple, Roasted Chicken. The Manager wants a diet with maximum cost. The diet chart is as follows:

**Table:** 

Nutrition	Banana	Apple	Roasted chicken	Minimum nutrition required
Calories	(5,10,13,14,18)	(1,2,3,4,5)	(2,6,8,10,14)	(2,11,23,34,45)
Protein	(3,4,5,6,7)	(1,5,6,7,11)	(1,4,5,9,16)	(10,47,52,65,76)
Carbohydrate	(3,6,9,12,15)	(2,5,7,8,8)	(1,1,1,1)	(3,18,56,76,87)
Maximum product required	(11,16,51,67,75)	(20,40,60,80,100)	(15,30,45,75,110)	

Now the problem is pentagonal neutrosophic number. Therefore, the decision-maker considers the confirmation degree of pentagonal number is (0.9, 0.1, 0.1). Based on step 2, we convert the above problem into pentagonal neutrosophic numbers in the following format.

$$ \hbox{max} = (11,16,51,67,75)x_{1} + (20,40,60,80,100)x_{2} + (15,30,45,75,110)x_{3} $$

Subject to,

$$ \begin{aligned} (5,10,13,14,18)x_{1} + (1,2,3,4,5)x_{2} + (2,6,8,10,14)x_{3} \le (2,11,23,34,45) \hfill \\ (3,4,5,6,7)x_{1} + (1,5,6,7,11)x_{2} + (1,4,5,9,16)x_{3} \le (10,47,52,65,76) \hfill \\ (3,6,9,12,15)x_{1} + (2,5,7,8,8)x_{2} + (1,1,1,1)x_{3} \le (3,18,56,76,87) \hfill \\ \end{aligned} $$

$$ x_{1} ,x_{2} ,x_{3} \ge 0 $$

By utilizing our step-3, new ranking function, the issue of PNLP problem is converting into crisp LP problem and the problem will be as follows:

$$ \hbox{max} = 39.6x_{1} + 54x_{2} + 47.7x_{3} $$

Subject to,

$$ \begin{aligned} 10.8x_{1} + 2.7x_{2} + 7.2x_{3} \le 20.7 \hfill \\ 4.5x_{1} + 5.4x_{2} + 6.3x_{3} \le 45 \hfill \\ 8.1x_{1} + 5.4x_{2} + 0.72x_{3} \le 43.2 \hfill \\ \end{aligned} $$

$$ x_{1} ,x_{2} \ge 0 $$

Here, we can solve the crisp LP problem by using LATEX 18.0 we get the optimal solution.

The solution is: $x_{1} = 0,x_{2} = 7.68,x_{3} = 0$ and $Z = 414$.

## Conclusion

In this article, we consider a pentagonal neutrosophic linear programming problem and solved it. We proposed a new ranking function for converting into pentagonal neutrosophic numbers to its equivalent crisp numbers. After using this ranking function for converting the problem to its crisp values, then we solve the problem by using any standard method. Using the proposed method, real life modelling of PNLP optimization could be easier and it may be easy to implement from the computational point of view. For the purpose of validation, We illustrated a simple diet optimization problem and two simple problems with pentagonal neutrosophic linear programming problem. By comparing our proposed model with other existing fuzzy models, we concluded that our proposed model is simpler, efficient and achieve better results than other researchers.

Further, researchers can fruitfully implement the idea of PNN based linear programming strategy in different research arenas like structural modeling, diagnostic problems, realistic modeling, project management and designing, linear fractional programming problem, transportation problem, Assessment problem, real worlds engineering problems, interval-valued fractional optimization problems, selection and recruitment based problems, image processing, fault detection based issues, pattern recognition etc.
